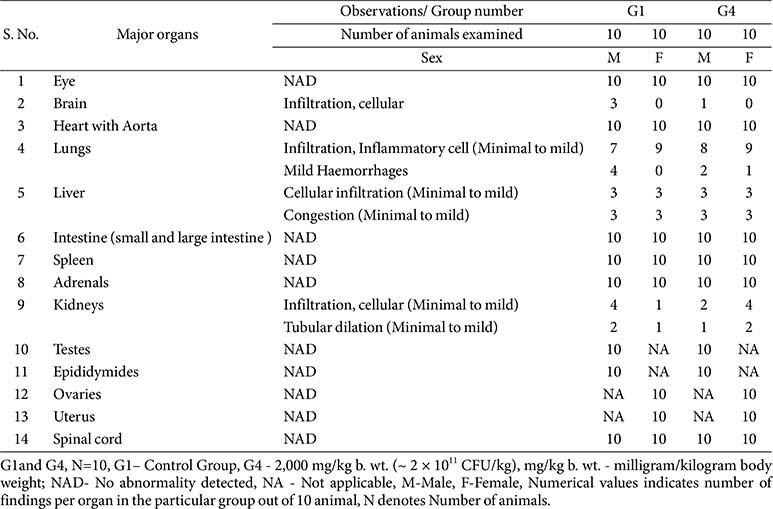# *In Vitro* and *In Vivo* Safety Evaluation of *Streptococcus salivarius* eK12, a Genetically Modified Dietary Probiotic Derived from the Oral Probiotic *S. salivarius* K12

**DOI:** 10.4014/jmb.2509.09016

**Published:** 2025-12-09

**Authors:** Francesco Di Pierro, Gayathri Veeraraghavan, Kuppusamy Kalaiselvan, Bahrudeen Ashif, Logeshwari Jeyakumar, Gopikrishnan Gopalakrishnan, Massimiliano Cazzaniga, Alexander Bertuccioli, Maria Laura Tanda, Nicola Zerbinati, Ikram Ujjan, Ali Akbar Bugti, Aasiya Bano, Yasmeen Gull, Nasrin Mumtaz, Amjad Khan

**Affiliations:** 1 Velleja Research, Scientific Department, Milano, Italy; 2 Department of Medicine and Technological Innovation, University of Insubria, Varese, Italy; 3 Microbiota International Clinical Society, Turin, Italy; 4 Centre For Toxicology and Developmental Research (CEFTE), Sri Ramachandra Institute of Higher Education and Research (DU), Porur, Chennai, India; 5 Diligence Bio Labs, Pondicherry, India; 6 Department of Biomolecular Sciences, University of Urbino Carlo Bo, Urbino, Italy; 7 Endocrine Unit, Department of Medicine and Surgery, University of Insubria, Varese, Italy; 8 Department of Pathology, Liaquat University of Medical and Health Sciences (LUMHS), Jamshoro, Pakistan; 9 Centre for Inflammation Research, University of Edinburgh, UK; 10 Research & Innovation Unit, National Emergency Operation Centre (NEOC), Islamabad, Pakistan; 11 DHQ Hospital, Turbat, Pakistan; 12 Jam Ghulam Civil Hospital, Hub, Pakistan; 13 Nuffield Division of Clinical Laboratory Sciences (NDCLS), Radcliffe Department of Medicine, University of Oxford, UK

**Keywords:** *Streptococcus salivarius* eK12, probiotic safety, ames test, acute oral toxicity, sub-chronic toxicity, food safety, genetic modification

## Abstract

Group A *Streptococcus* (GAS) pharyngitis is a common and recurrent childhood illness, usually treated with antibiotics. While effective, repeated antibiotic use contributes to antimicrobial resistance, disrupts host microbiota, and increases adverse effects. *Streptococcus salivarius* K12 is an extensively studied probiotic for oral health, particularly for reducing the incidence of GAS pharyngotonsillitis. A modified version, *S. salivarius* eK12, has recently been developed from the wild-type K12 through genetic modification to enhance anti-GAS activity. However, its safety and tolerability have not previously been evaluated, and this study provides the first systematic assessment of eK12 under OECD-guided toxicological frameworks. Genotoxicity was assessed through the bacterial reverse mutation assay (OECD 471, Ames test) in five *Salmonella typhimurium* strains, with and without metabolic activation. Acute oral toxicity was examined in female Wistar rats at a limit dose of 2,000 mg/kg (~2 × 10^11^ CFU/kg) (OECD 423). Sub-chronic safety was investigated in a 90-day repeated-dose oral toxicity study (OECD 408) using Sprague Dawley rats randomized to vehicle, low, mid, or high eK12 dose groups, with additional recovery cohorts (vehicle, high dose) observed for 28 days post-treatment. Results showed no mutagenic activity, no mortality, and no treatment-related abnormalities in clinical, hematological, biochemical, or histopathological parameters. Minor fluctuations were incidental and non–dose dependent. Recovery groups confirmed the absence of delayed or persistent toxicity. These findings indicate that eK12 is potentially non-mutagenic, non-toxic, and well tolerated, with a NOAEL of 2,000 mg/kg/day (~2 × 10^11^ CFU/kg/day), retaining the favorable safety profile of its parental strain K12 and supporting its further development.

## Introduction

Recurrent Group A *Streptococcus* (GAS) throat infections in children remain a persistent clinical challenge, managed with antibiotics [1]. However, repeated or prolonged antibiotic use is associated with several important drawbacks, including the development of antimicrobial resistance, adverse drug reactions, and disruption of gut microbiota [2-4]. In some cases, antibiotic therapy may fail to completely eradicate GAS from the oropharynx, resulting in persistent carrier states or recurrent infections [5, 6]. Prolonged antibiotic use has also been associated with an increased risk of secondary infections and potential impairment of host immune competence [7, 8]. These limitations have generated growing interest in alternative preventive approaches such as precision probiotics and bacterial replacement therapy [9].

*Streptococcus salivarius* is a naturally occurring commensal bacterium that colonizes the oral cavity, contributing to microbial homeostasis and upper respiratory tract health [10, 11]. *S. salivarius* K12 (LMG P-27407), a bacteriocin-producing and γ-interferon–inducing probiotic first isolated in 1989 from a child’s tongue [12, 13], has been shown to reduce the incidence of recurrent GAS pharyngitis, acute otitis media, PFAPA (Periodic Fever, Aphthous Stomatitis, Pharyngitis, and Cervical Adenitis), and viral upper respiratory tract infections, including SARS-CoV-2, in both children and adults [14-22]. *S. salivarius* K12 is registered as a food supplement across Europe, Australia, Asia, and the Americas, and holds GRAS (Generally Recognized as Safe) status in the United States, reflecting its well-established safety record in both children and adults [11, 23, 24]. Evidence suggests that *S. salivarius* K12 inhibits the growth of pathogens such as *S. pyogenes*, *S. pneumoniae*, and other pharyngeal bacteria through competitive exclusion and the production of antimicrobial peptides, including the lantibiotics salivaricin A2 (2,368 Da) and salivaricin B (2,740 Da) [25-27]. The ability of *S. salivarius* K12 to produce a variety of bacteriocin-like inhibitory substances (BLIS), with activity that appears to be particularly effective against GAS bacteria, is of particular therapeutic interest for protection against pathogenic GAS infection in the oral cavity, as both have adapted to a principally oral mucosal existence in humans. By colonizing the oral mucosa, *S. salivarius* K12 forms a protective barrier against pathogens, preventing their adhesion and subsequent colonization. Accordingly, supplementation with *S. salivarius* K12 may help decrease the recurrence and severity of GAS infections in children, providing a supportive strategy that could reduce reliance on antibiotics and mitigate risks of resistance and microbiota imbalance [28-30].

Despite its beneficial effects, *S. salivarius* K12 has shown limitations under specific conditions. Co-colonization studies in mice and a human saliva model revealed that *S. pyogenes* can exploit quorum-sensing pathways triggered by K12 via the *nip* gene, leading to premature release of the SpeB protease and inactivation of salivaricins A2 and B [31]. To overcome these limitations while retaining the probiotic attributes of the parent strain, a genetically modified version, known as *S. salivarius* eK12, has been recently developed from K12. In this variant, silencing of the *nip* gene and introduction of a strong PtufA promoter upstream of the plasmid-borne *sarBGC* gene, responsible for producing salivabactin, restored and enhanced antagonism, enabling effective elimination of *S. pyogenes*.

Although these genetic modifications enhanced antimicrobial functionality in strain eK12, they also introduced uncertainties regarding genetic stability, unintended metabolic effects, and host–microbe interactions. To explore these aspects, the present study evaluated the safety of *S. salivarius* eK12 under OECD guideline–compliant toxicological frameworks. Three complementary assays were undertaken: a bacterial reverse mutation test (OECD 471, Ames test) to assess genotoxic potential; an acute oral toxicity study in rats (OECD 423) to evaluate immediate systemic effects; and a 90-day repeated-dose oral toxicity study (OECD 408) to examine longer-term safety, potential target organ effects, and overall tolerability [24, 32-34]. All studies were performed under Good Laboratory Practice (GLP) conditions to ensure data quality and provide supportive evidence regarding the safety of *S. salivarius* eK12.

## Materials and Methods

### *S. salivarius* eK12 Strain

The *S. salivarius* eK12 strain used in this study was a lyophilized formulation with a pale-yellow crystalline appearance, containing a viable cell count of 1.0 × 10^11^ CFU/g. The strain deposited at the Ghent collection with ID number LMG P-33696, was manufactured by Teracell s.r.l., Italy. All Critical reagents/strains details are procured from authorised vendors, including TA strains from Moltox, USA, Biochemical analysis QC and kits from BioSystems, T3, T4 and TSH ELIZA kit from Kirshgen BioSystems, Electrolyte QC from Cobas, M-52 LH and Diff Lyse from Mindray. All other chemicals/reagents were of analytical grade.

### Study design

Three toxicological studies were performed to evaluate the safety profile of *S. salivarius* eK12. These included bacterial reverse mutation assay (Ames Test), an acute oral toxicity study, and a sub-chronic 90-day repeated dose oral toxicity study.

### Bacterial Reverse Mutation Assay (Ames Test)

The mutagenic potential of *S. salivarius* eK12 was evaluated using the bacterial reverse mutation assay in accordance with OECD test guideline 471 (1997, corrected 2020) under GLP compliance at Diligence Bio Laboratories, Pondicherry, India [33]. The study was approved by the Institutional Biosafety Committee of the test facility (Approval No. DB/IBSC/05/11/24), and biosafety practices were followed according to national guidelines.

The assay assessed the ability of eK12 to induce point mutations in the histidine operon of five *Salmonella typhimurium* tester strains (TA98, TA100, TA1535, TA1537, and TA102), both in the presence and absence of a metabolically active rat-liver S9 microsomal fraction. Distilled water served as the vehicle control, while strain-specific mutagens were used as positive controls: sodium azide for TA100 and TA1535, 2-nitrofluorene for TA98, 9-aminoacridine for TA1537, mitomycin-C for TA102, and benzo[a]pyrene for all strains with metabolic activation.

A preliminary cytotoxicity range-finding test confirmed suitability of eK12 concentrations up to 5 mg/plate (5 × 10^8^ CFU/plate). The main mutagenicity assessment comprised two independent trials using the plate incorporation method. In Trial I, assays were conducted with and without metabolic activation (5% S9), while Trial II served as a confirmatory test with 10% S9. eK12 was tested at five concentrations (0.3125–5 mg/plate)(equivalent to 3.91 × 10^6^ to 5 × 10^8^ CFU/plate), with all treatments and controls performed in triplicate. Plates were incubated at 37°C for 48 h, and revertant colonies were manually counted. Mutagenicity was defined as a dose-dependent or ≥2-fold increase in revertant counts relative to vehicle controls, confirmed by reproducibility and appropriate positive control responses. All test preparations were freshly prepared in distilled water and adjusted to physiological pH (6.8–7.4) prior to plating. Vehicle and controls were included to verify that no interference arose from pH variation, antibiotic residues, or formulation excipients. These procedures were performed in accordance with OECD 471 requirements to ensure assay validity and reproducibility.

### Acute Oral Toxicity Study

The acute oral toxicity of *S. salivarius* eK12 was evaluated in female Wistar rats in accordance with OECD test guideline 423 (2001) under GLP compliance at Diligence Bio Laboratories, Pondicherry, India [32]. Ethical approval was obtained from the Institutional Animal Ethics Committee (IAEC) (Approval No. DB/IAEC/AORT/Bactoblis EVOL, *S. salivarius* eK12)/004/11-24) and the Institutional Biosafety Committee of the test facility, with all procedures conducted in accordance with CPCSEA animal welfare guidelines.

Young, nulliparous, non-pregnant female Wistar rats (9 weeks old) were selected and acclimatized prior to dosing. Animals were housed under controlled environmental conditions with free access to food and water. Test formulations were freshly prepared in distilled water and administered by oral gavage at a fixed dose of 2,000 mg/kg (~2 × 10^11^ CFU/kg) body weight, the OECD limit dose. Two sequential groups of three rats each (*n* = 6 total) were treated according to the stepwise procedure described in OECD 423.

Rats were fasted before dosing and monitored for 14 days following administration. Observations included mortality, morbidity, and clinical signs of toxicity, with particular attention to the first 4 h post-dosing and daily thereafter. Body weights were recorded on days 0, 7, and 14. At study termination, all animals were euthanized and subjected to gross necropsy to assess for pathological changes.

### 90-Day Repeated Dose Oral Toxicity Study

A 90-day repeated-dose oral toxicity study of *S. salivarius* eK12 was conducted in Sprague Dawley rats in accordance with OECD test guideline 408 (2018) under GLP compliance at the Centre for Toxicology and Developmental Research (CEFTE), Sri Ramachandra Institute of Higher Education and Research, Chennai, India [34]. Ethical approval was obtained from the Institutional Animal Ethics Committee (Approval No. IAEC/75/SRIHER/943/2024) and the Institutional Biosafety Committee.

A total of 100 rats (6–8 weeks old; 50 males, 50 females) were randomized into four main groups G1, G2, G3, and G4 (*n* = 20/group; 10/sex) and two recovery groups G1R and G4R (*n* = 10/group; 5/sex) (Table 1). The main study groups received vehicle control (purified water only) (G1), low dose eK12 500 mg/kg (~0.5 × 10^11^ CFU/kg) (G2), mid dose eK12 1,000 mg/kg (~1 × 10^11^ CFU/kg) (G3), or high dose eK12 2,000 mg/kg (~2 × 10^11^ CFU/kg) (G4) once daily by oral gavage for 90 days. Recovery groups included a vehicle control (G1R) and a high-dose group (2,000 mg/kg), (G4R), observed for an additional 28 days post-treatment to assess reversibility, persistence, or delayed effects.

Animals were monitored daily for mortality and clinical signs, with weekly assessments of detailed clinical observations, body weight, and food consumption. Ophthalmological examinations, functional observation battery tests, and neurological assessments were performed during the study. Hematology, clinical chemistry, electrolytes, thyroid hormones, and urinalysis were assessed at scheduled intervals (day 91 for main groups and day 119 for recovery groups). At termination, all animals underwent necropsy with organ weight measurements and histopathological examination of major organs and tissues.

Data were analyzed using one-way ANOVA with Dunnett’s post hoc test for main groups and Student’s *t*-test for recovery groups, with significance set at *p* ≤ 0.05, and are presented as mean ± standard deviation (SD) values using Sigma Plot 14.5 software. All hematological, biochemical, and organ-weight measurements were obtained using calibrated equipments/instruments and certified reference standards, and values are expressed in absolute units (*e.g.*, mg/dL, g/dL, U/L, mmol/L, %) in compliance with OECD and GLP data-reporting requirements.

## Results

### Bacterial Reverse Mutation Assay (Ames Test)

No cytotoxicity or precipitation of *S. salivarius* eK12 was observed at any concentration in the preliminary range-finding test with *S. typhimurium* strains TA98 and TA100, confirming suitability of the selected doses for the main assay.

**Fig. 1:**
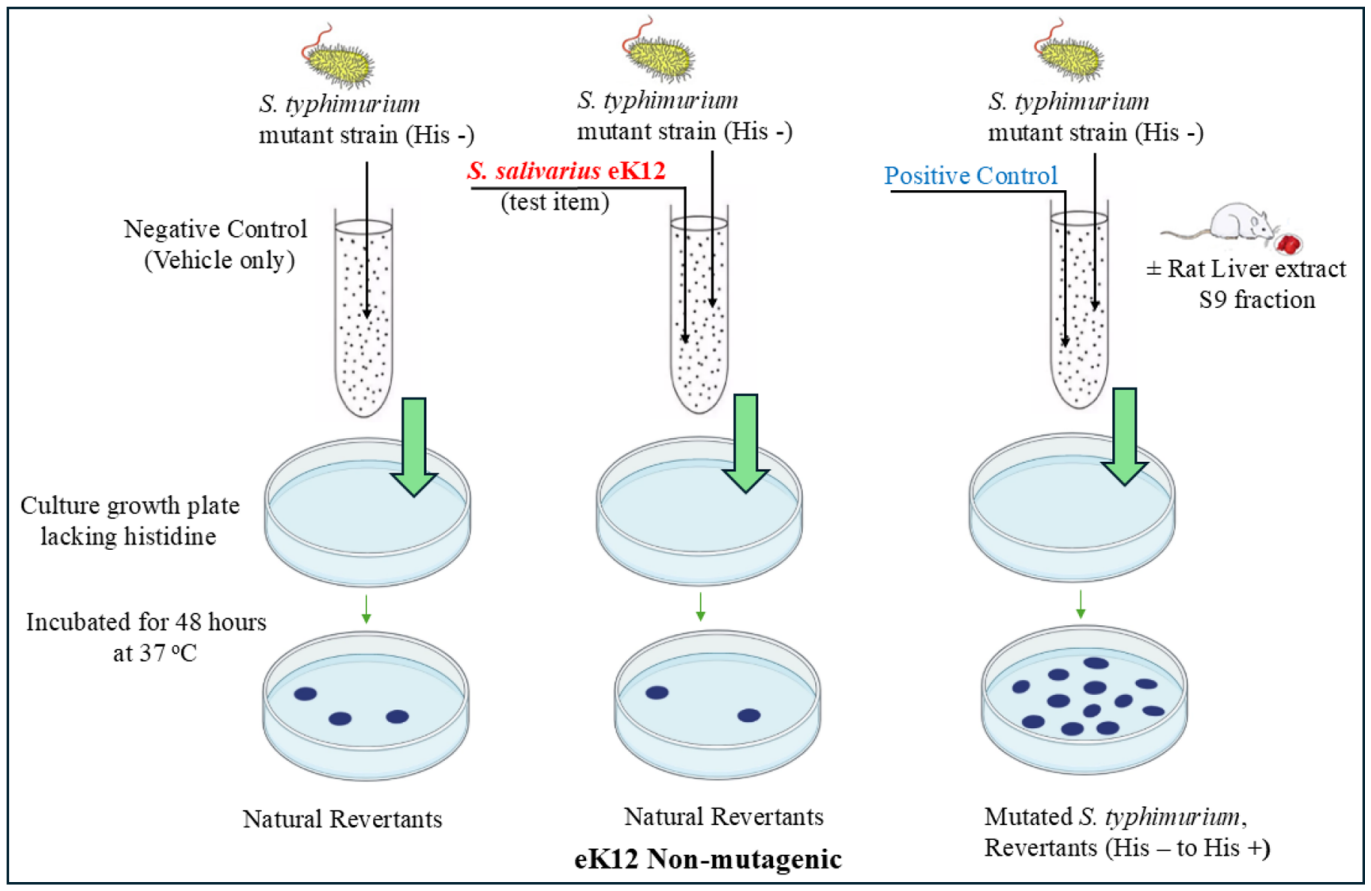
Illustration of the bacterial reverse mutation test (Ames test) (OECD 471) to investigate the potential mutagenic or genotoxic effect of *S. salivarius* eK12. eK12 was tested at five concentrations (0.3125–5 mg/plate) (equivalent to 3.91 × 10^6^ to 5 × 10^8^ CFU/plate), with all treatments and controls performed in triplicate.

Across both Trial I and Trial II, eK12 did not induce any biologically relevant increase in revertant colonies in any of the five tester strains (TA98, TA100, TA1535, TA1537, TA102), either with or without metabolic activation (S9 mix) (Fig. 1). The mean revertant counts for all treated groups remained comparable to vehicle controls and within historical ranges. No dose-response trend or reproducible increases were detected.

All positive controls produced clear and statistically significant increases in revertant colonies, demonstrating the validity and sensitivity of the assay. According to OECD test guideline 471, mutagenicity is defined as a ≥2-fold increase in revertants for TA98, TA100, and TA102, or a ≥3-fold increase for TA1535 and TA1537. No such responses were observed with eK12 under any condition.

Based on these findings, *S. salivarius* eK12 was considered non-mutagenic in the bacterial reverse mutation assay.[Fig F2]

### Acute Oral Toxicity Study

All six female Wistar rats survived the 14-day observation period following a single oral administration of *S. salivarius* eK12 at 2,000 mg/kg (~2 × 10^11^ CFU/kg) (Fig. 1). No mortality, morbidity, or treatment-related clinical signs of toxicity were observed in either dosing step. Animals remained active and healthy throughout the study.

Body weight measurements on Days 0, 7, and 14 showed normal progression with no deviations from expected growth trends. At necropsy, no macroscopic abnormalities were observed on external or internal examination. There were no indications of systemic or organ-specific toxicity.

Based on the absence of adverse clinical signs, mortality, or gross pathological findings, the median lethal dose (LD_50_) of *S. salivarius* eK12 was estimated to be greater than 2,000 mg/kg (~2 × 10^11^ CFU/kg) in female Wistar rats. According to OECD test guideline 423 and the Globally Harmonized System of Classification and Labelling of Chemicals (GHS, 10^th^ Revised Edition, 2023), *S. salivarius* eK12 was categorized as GHS category 5 or unclassified for acute oral toxicity.

### 90-Day Repeated Dose Oral Toxicity Study

**Fig. 3:**
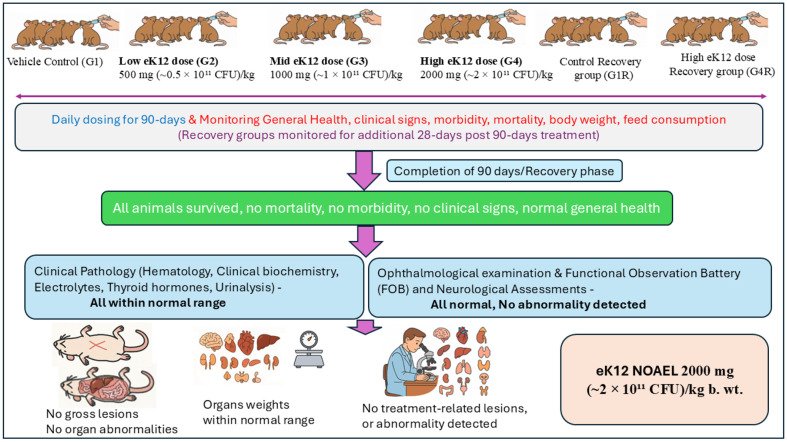
Schematic representation of the 90-day repeated dose oral toxicity study of *S. salivarius* eK12 in albino Wistar rats in accordance with OECD test guideline 408 under GLP compliance.

Results of the 90-day repeated dose oral toxicity studies are summarised in Fig. 3.

**General health, clinical signs, and mortality:**

No mortality or morbidity was observed during the 90-day dosing period or 28-day recovery phase. Daily cage-side and weekly detailed clinical observations did not reveal treatment-related abnormalities in any group (G1–G4, G1R, G4R).

**Body weight and feed consumption:**

Body weight gains were comparable between treated and control groups (Table 2). Transient fluctuations, including decreased weight gain in males (G3, G4) and increased gain in females (G2–G4), were not dose-dependent, not sustained, and unaccompanied by changes in feed intake. These were considered incidental.

**Ophthalmology and functional observations:**

No ocular changes were detected pre-dose or at study termination. Neurological assessments, including sensory reactivity, grip strength, and motor activity, were within normal limits in all groups (Table 3).

**Hematology:**

No dose-related effects were observed. Sporadic significant changes (*e.g.*, reduced eosinophils, prolonged clotting time, altered RBC indices or WBC counts) were within reference ranges, lacked dose–response relationships, and had no pathological correlates; thus, they were considered incidental (Tables 4 and 5).

**Clinical biochemistry, electrolytes, and thyroid hormones:**

Biochemical variations (*e.g.*, protein, albumin, cholesterol, triglycerides, AST) reached statistical significance in some groups but were inconsistent, reversible, and within physiological ranges (Tables 6 and 7). Electrolyte values were normal, with a single non-persistent increase in serum phosphorus in G3 females (Table 8). Thyroid hormones showed marginal fluctuations without histopathological correlates (Table 8).

**Urinalysis, gross pathology, and organ weights:**

No treatment-related changes were observed in urine parameters or at necropsy (Tables 9 and 10). Organ weight differences (*e.g.*, adrenals in G3 males, heart in G4R males, pituitary in G4R females) were within historical control ranges, not dose-dependent, and unaccompanied by histological changes (Table 11).

**Histopathology:**

Microscopic examination was performed on major organs including the brain, eyes, heart with aorta, lungs, liver, intestines, spleen, adrenals, kidneys, testes, epididymides, ovaries, uterus, and spinal cord (Table 12). No treatment-related lesions were identified. Minimal to mild changes, such as inflammatory cell infiltration in the brain, lungs, liver, and kidneys, mild pulmonary hemorrhages, hepatic congestion, and sporadic tubular dilation in the kidneys, were observed at comparable frequencies in both control (G1) and high-dose (G4) groups. These findings are consistent with spontaneous or incidental background changes in Sprague Dawley rats and were not considered toxicologically significant.

## Discussion

This study provides the first comprehensive safety evaluation of the genetically modified probiotic *S. salivarius* eK12 using OECD guideline–based toxicological studies. The investigations included mutagenicity testing, acute oral toxicity, and a 90-day repeated-dose oral toxicity study with an additional 28-day recovery phase to evaluate potential delayed or reversible effects. Collectively, the results demonstrate that eK12 is safe and well tolerated, establishing a NOAEL of 2,000 mg/kg/day (~2 × 10^11^ CFU/kg/day) in rats.

The Ames test confirmed that genetic engineering of K12 did not introduce mutagenic potential, consistent with the well-established safety profile of wild-type *S. salivarius* K12 [14-19, 21, 22, 24, 35]. The alignment of results between eK12 and its parent strain indicates that the modifications introduced to overcome GAS evasion mechanisms—such as silencing the *nip* gene, enhancing salivabactin production, and protecting bacteriocins from proteolytic degradation—did not alter its intrinsic genomic safety. The absence of mutagenicity supports the genomic stability of the engineered strain and aligns with international recommendations such as EFSA and WHO for genetically modified microbial safety evaluation.

The acute oral toxicity study showed that eK12 did not elicit adverse effects at the OECD 423 limit dose of 2,000 mg/kg (~2 × 10^11^ CFU/kg/day), corresponding to GHS category 5 or unclassified for acute oral toxicity. These findings parallel those previously reported for wild-type K12, indicating that the genetic modifications did not alter fundamental tolerability [35].

The 90-day repeated-dose study further reinforced the safety of eK12. No mortality, morbidity, or treatment-related clinical signs were observed, and growth, feed consumption, functional neurological outcomes, ophthalmology, and urinalysis remained unaffected. Hematologic and biochemical variations were sporadic, lacked dose dependence, and were unaccompanied by pathological correlates. Organ weight changes were minor and inconsistent, while histopathology revealed only minimal background changes that occurred in both treated and control animals. Importantly, the inclusion of a recovery phase demonstrated no persistence or delayed toxicity, with transient changes normalizing after treatment cessation. No treatment-related effects were observed across the dose range (0.5–2 × 10^11^ CFU/kg/day) or at any time point up to 118 days, confirming the absence of dose- or time-dependent toxicity.

Taken together, these findings establish a NOAEL of 2,000 mg/kg/day (~2 × 10^11^ CFU/kg/day) in both sexes, representing a safety margin more than 10,000 times greater than the typical daily intake of *S. salivarius* K12 as a probiotic (one billion CFU). The results are consistent with the established safety record of the parent strain, confirming that the modifications in eK12 did not compromise its intrinsic safety profile [24]. To our knowledge, this is the first comprehensive safety evaluation of an engineered *S. salivarius* strain, supporting its further development as a potential non-antibiotic protective strategy for recurrent GAS infections in the broader effort to reduce antimicrobial resistance.

Engineered probiotics such as eK12 represent an emerging class of live biotherapeutics designed to address limitations of conventional strains. While traditional probiotics like K12 are well established as safe, their efficacy can be constrained by pathogen countermeasures, as demonstrated in co-colonization studies with GAS [31]. The rational modification of K12 to create eK12 illustrates how synthetic biology can enhance antimicrobial activity without compromising safety. More broadly, this aligns with the development of next-generation probiotics [36, 37], which aim to combine established probiotic benefits with engineered functionalities for targeted infection control and antimicrobial resistance mitigation. The present findings provide reassurance that such modifications can be achieved safely and support the translational development of engineered probiotics.

Future studies should include human clinical trials, particularly in pediatric populations, to confirm safety and tolerability in the intended target group. Additional molecular analyses, such as metabolomic and proteomic profiling, may further exclude unintended off-target effects. In addition, future investigations should include targeted genetic and biochemical studies to validate the mechanisms associated with the enhanced anti–*Streptococcus pyogenes* activity of *S. salivarius* eK12 and to ensure that the introduced modifications do not influence unrelated physiological or host-interaction pathways. Furthermore, future mechanistic and formulation studies should evaluate the stability and biological activity of *S. salivarius* eK12 preparations under physiologic pH, ionic strength, and serum conditions to further confirm relevance to in-vivo environments. Nevertheless, the present findings provide a comprehensive safety foundation indicating that the rational modification of *S. salivarius* K12 to enhance anti-GAS activity has preserved its inherent safety.

## Conclusion

*S. salivarius* eK12 was shown to be non-mutagenic, non-toxic at acute high doses, and safe during 90 days of repeated oral administration in rats, with a NOAEL of 2,000 mg/kg/day (~2 × 10^11^ CFU/kg/day). No treatment-related clinical, hematologic, biochemical, or histopathological changes were observed, and findings during a 28-day recovery phase confirmed the absence of persistent or delayed effects. Importantly, safety was demonstrated at exposure levels more than 10,000 times greater than the typical daily intake of *S. salivarius* K12 as a probiotic, indicating a wide margin of safety. These results show that the genetic modifications introduced to enhance anti-GAS activity did not compromise the strain’s intrinsic safety profile. Collectively, this study provides the first comprehensive evidence supporting the safety of an engineered *S. salivarius* strain and establishes a foundation for future therapeutic benefits evaluation.

## Figures and Tables

**Fig. 2 F2:**
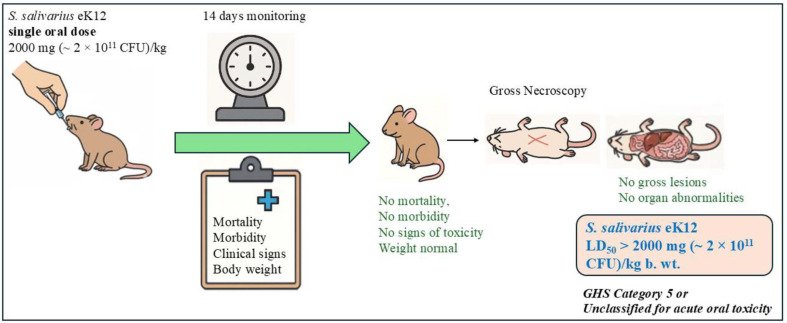
Schematic representation of the oral acute toxicity study of *S. salivarius* eK12 in female Wistar rats in accordance with OECD test guideline 423 (2001) under GLP compliance.

**Table 1:** Study design and *S. Salivarius* eK12 dosing regimen -90-day repeated dose oral toxicity study.

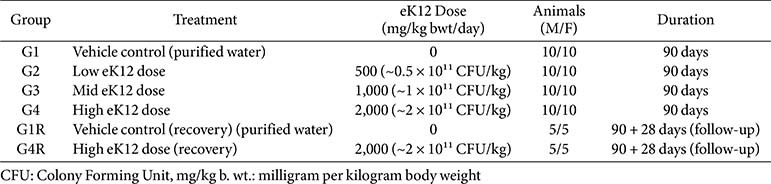

**Table 2:** Weekly body weight values – main and recovery groups.

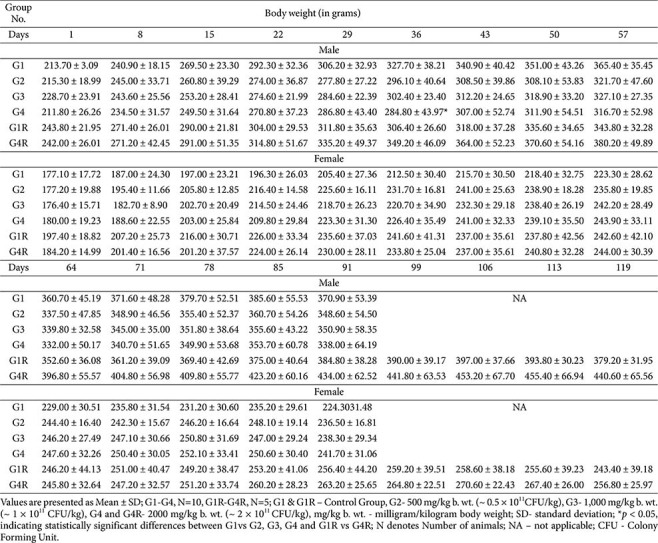

**Table 3:** Functional observation battery results –motor activity and grip strength.

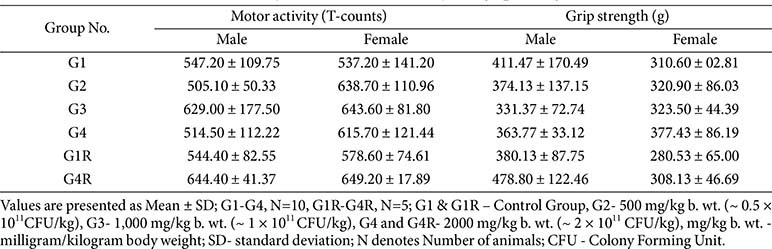

**Table 4:** Hematology results –main (day 91) and recovery group (day 119) -male.

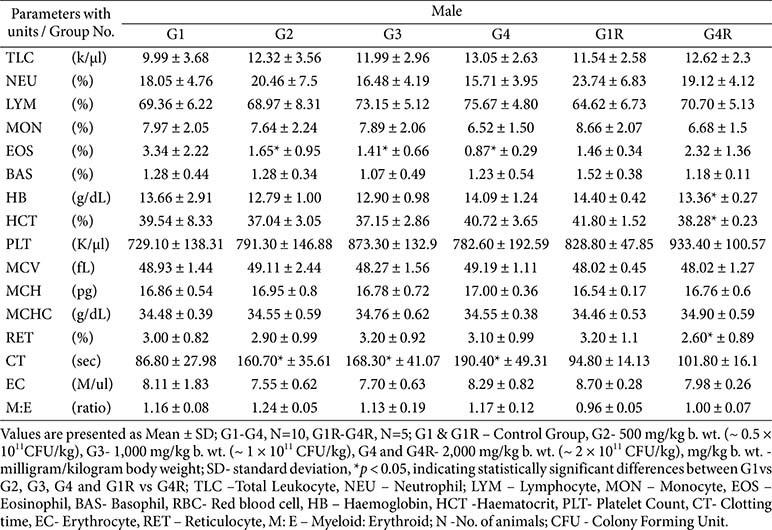

**Table 5:** Hematology results –main (day 91) and recovery group (day 119) - female.

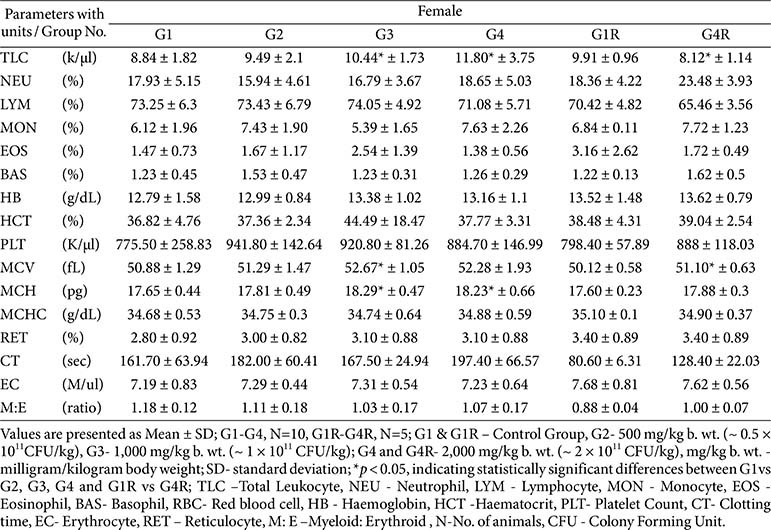

**Table 6:** Biochemistry parameters - main (day 91) and recovery group (day 119)-male.

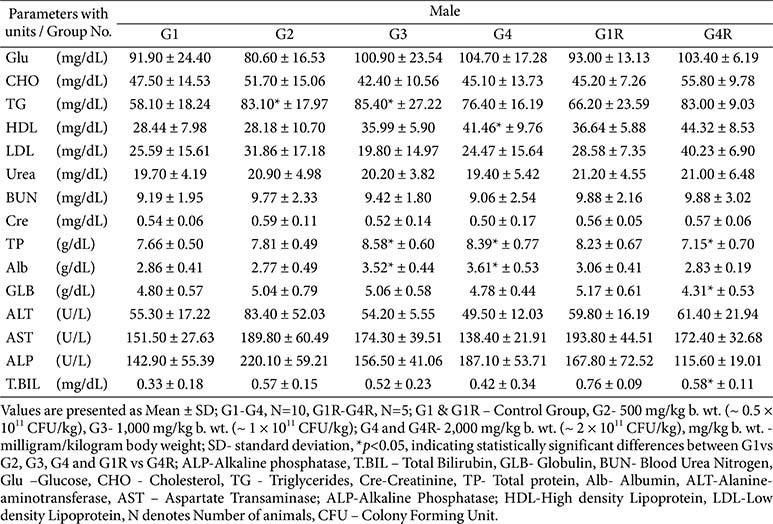

**Table 7:** Biochemistry parameters - main (day 91) and recovery group (day 119) -female.

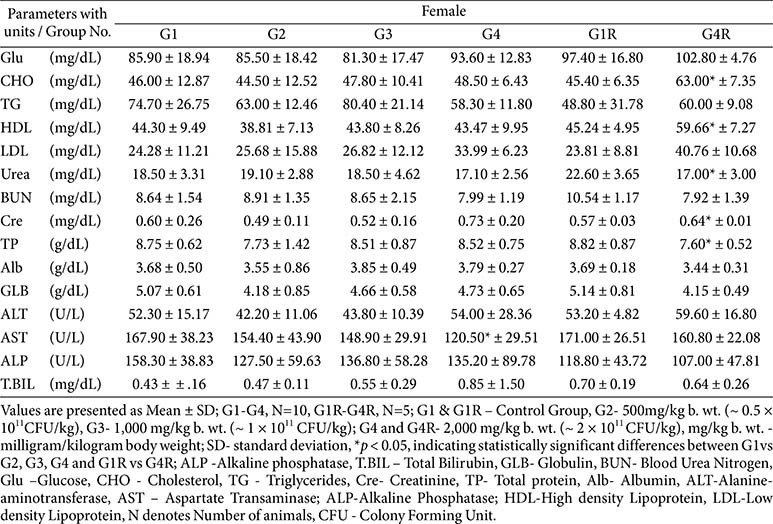

**Table 8:** Serum electrolytes and thyroid hormones results - main (day 91) and recovery group (day 119).

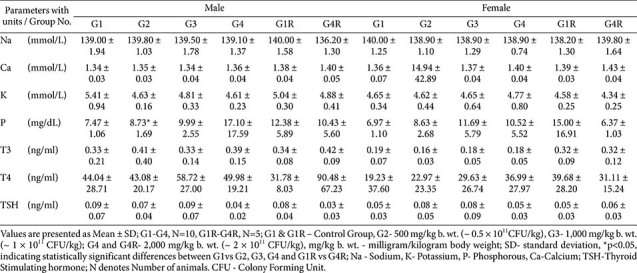

**Table 9:** Summary of gross pathology-main and recovery group- main (day 91) and recovery group (day 119).

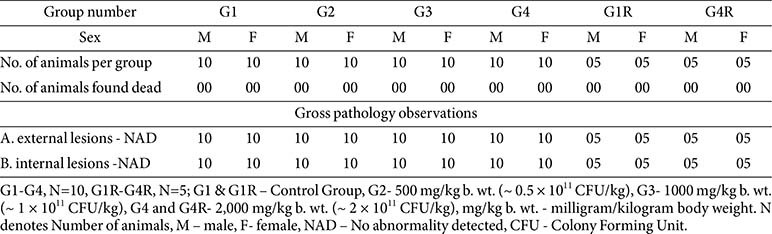

**Table 10:** Summary absolute organ weight – male (main and recovery group).

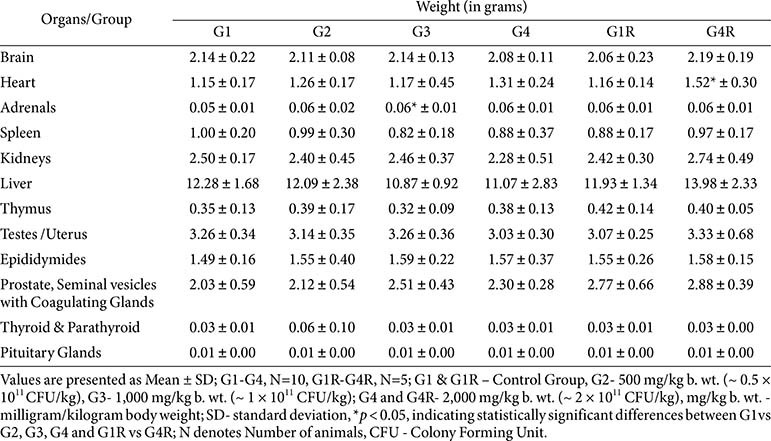

**Table 11:** Summary absolute organ weight – female (main and recovery group).

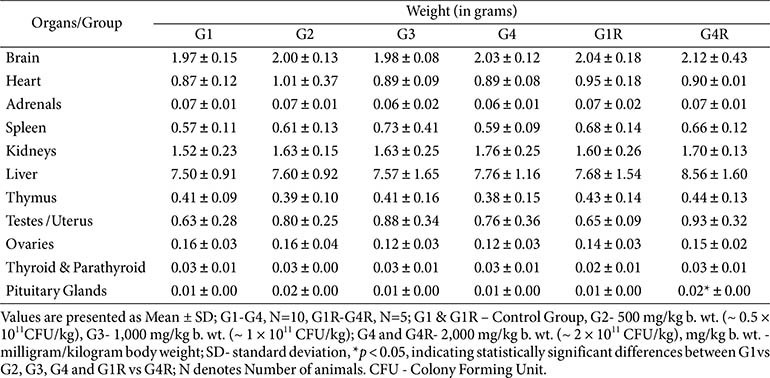

**Table 12:** Summary of histopathology findings.